# Elbow dislocation approach for complex elbow fractures: a cadaveric study

**DOI:** 10.1186/s13018-023-04478-x

**Published:** 2023-12-20

**Authors:** Yang Liu, Yuling Gao, Xiaopei Xu, Yanrui Zhao, Hanzhou Wang, Qingnan Sun, Binzhi Zhao, Siyuan Wang, Junlin Zhou

**Affiliations:** 1 Affiliated Beijing Chaoyang Hospital of Capital Medical University, Bejing, China; 2 Beijing Chaoyang Hospital, Capital Medical University, Gongtinan Road 8#, Beijing, 100020 China

**Keywords:** Complex elbow fracture, Surgical approach, Olecranon osteotomy approach, Elbow dislocation, Distal humerus, Coronoid process of ulna, Cadaveric study, Articular exposure

## Abstract

**Background:**

Approach need to be considered when surgeons dealt with complex elbow injuries and the choice of the approach is a challenge for surgeons due to the complex anatomy. On the basis of releasing the lateral collateral ligament, we modified the dislocation technique to pursue the superior exposure including not only the distal humeral surface but also the anterior facet of the coronoid process.

**Methods:**

A total of 4 cadaver specimens and 8 elbows were included in the study. Each cadaver provided one elbow for either the elbow dislocation approach or the posterior olecranon approach. The exposed distal articular surface of humerus, humeral capitulum, humeral trochlea, anterior trochlea of distal humerus, posterior trochlea of distal humerus and the ulnar coronoid process surface were marked by image J software and calculated for a comparison for each surgical approach.

**Results:**

The total distal humeral surface was exposed as a median of 98.2 (97.6, 99.6)% and 62.0 (58.3, 64.5)% for the elbow dislocation approach and the olecranon osteotomy approach (*P* < 0.001), the capitulum 100% and 32.4 (28.0, 39.2)% (*P* < 0.001), the trochlea 93.2(90.1, 96.9)% and 72.5 (65.2, 78.8)% (*P* < 0.001), the anterior trochlear articular surface 96.0(93.0, 97.4)% and 50.3 (43.6, 59.1)% (*P* < 0.001), the posterior trochlear articular surface 95.4 (93, 100)% and 100% (*P* = 0.76) and the articular surface of the coronoid process of ulna 71.3 (66.0, 74.2)% and 0% (*P* < 0.001).

**Conclusion:**

For complex elbow fractures, the technique of elbow dislocation provides complete exposure of the distal humerus surface and a significant portion of the coronoid process surface, facilitating direct visualization for reduction and fixation.

*Level of evidence* Anatomy Study; Cadaver Dissection.

## Introduction

Approaches need to be considered when surgeons dealt with complex elbow injuries such as distal coronal plane fractures or terrible triad injuries because internal fixation has become the standard treatment for those injuries [1]. However, the choice of the approach is a challenge for surgeons due to the complex anatomy of the elbow. The surgeons have to balance the exposure of the fracture area and the protection of important anatomy structure, neurovascular and ligamentous.

Several approaches have been put forward for different kinds of elbow fractures [2]. For example, the lateral approach is recommended for radial head or capitulum fractures; the medial approach is recommended for anterior medial facet coronoid fractures; and the anterior approach is used to expose the anterolateral facet and the trochlea fractures; and the posterior approach like the olecranon osteotomy approach is widely used for the maximum exposure of the joint. Recently, Hoyt et al. [3] demonstrated, through cadaveric studies, that the lateral elbow dislocation method provides significantly higher exposure of 95.9% of the anterior surface and 100% of the capitulum compared to olecranon osteotomy. Several clinical case reports have indicated that this approach can effectively enhance exposure of the distal humeral articular surface [4–6]. The release of the lateral ligament became the key procedure to enlarge the visualization of the distal articular surface. This anatomic cadaveric study presents a technique for approaching the elbow structure based on Hoyt’s study and compares it with the approach of olecranon osteotomy. On the basis of releasing the lateral collateral ligament, we modified the dislocation technique to enhance the exposure encompassing not only the distal humeral surface but also the anterior facet of the coronoid process.

## Materials and methods

General data inclusion criteria were: (1) cadavers aged over 18 years old; (2) complete upper limb from shoulder to hand. Exclusion criteria were: (1) concomitant deformity or severe joint degeneration; (2) the deep structures of the cadaver have been dissected in any form or degree. The present study was granted approval by the Medical Ethics Committee of Beijing Chaoyang Hospital, and the experimental investigation involving cadavers adhered to the ethical standards outlined in the 1964 Declaration of Helsinki. A total of 4 cadaver specimens and 8 elbows were included in the study. Before the study, each arm was intact from the humeral capitulum to the hand, with no fractures, dislocations, soft tissue defects or any incisions on the limb. Each cadaver provided one elbow for either the lateral epicondylar dislocation approach or the posterior olecranon approach so that each elbow in each cadaver underwent a different approach with each surgical exposure group including 2 left and right elbows. Two members of the surgical team were assigned to each entry route under the guidance of an upper extremity trauma surgeon.

### Elbow dislocation surgical technique

After complete thawing, the cadaveric specimen was placed on the autopsy table in a lateral position with the elbow in flexion of approximately 135° and forearm pronation. The skin incision marking line was drawn with an oiling marker pen, and the skin incision was centered on the lateral epicondyle of the humerus. It extended from the anterior aspect of the lateral column of the distal humerus to a point 3 cm distal to the radial head, while proximally reaching 5 cm away from the lateral epicondyle, resulting in a surgical incision length of 8 cm long. The deep fascia was incised along the medial border of the brachioradialis muscle. At the level of the proximal elbow, the radial nerve was identified between the brachialis and brachioradialis muscles. The brachioradialis muscle was pulled laterally, while the brachialis muscle and the overlying biceps brachii muscle were pulled medially. A longitudinal incision was made anterior to the joint capsule between the radial nerve (lateral) and the brachialis muscle (medial) to expose the capitulum and lateral compartment of the elbow.

**Fig. 1:**
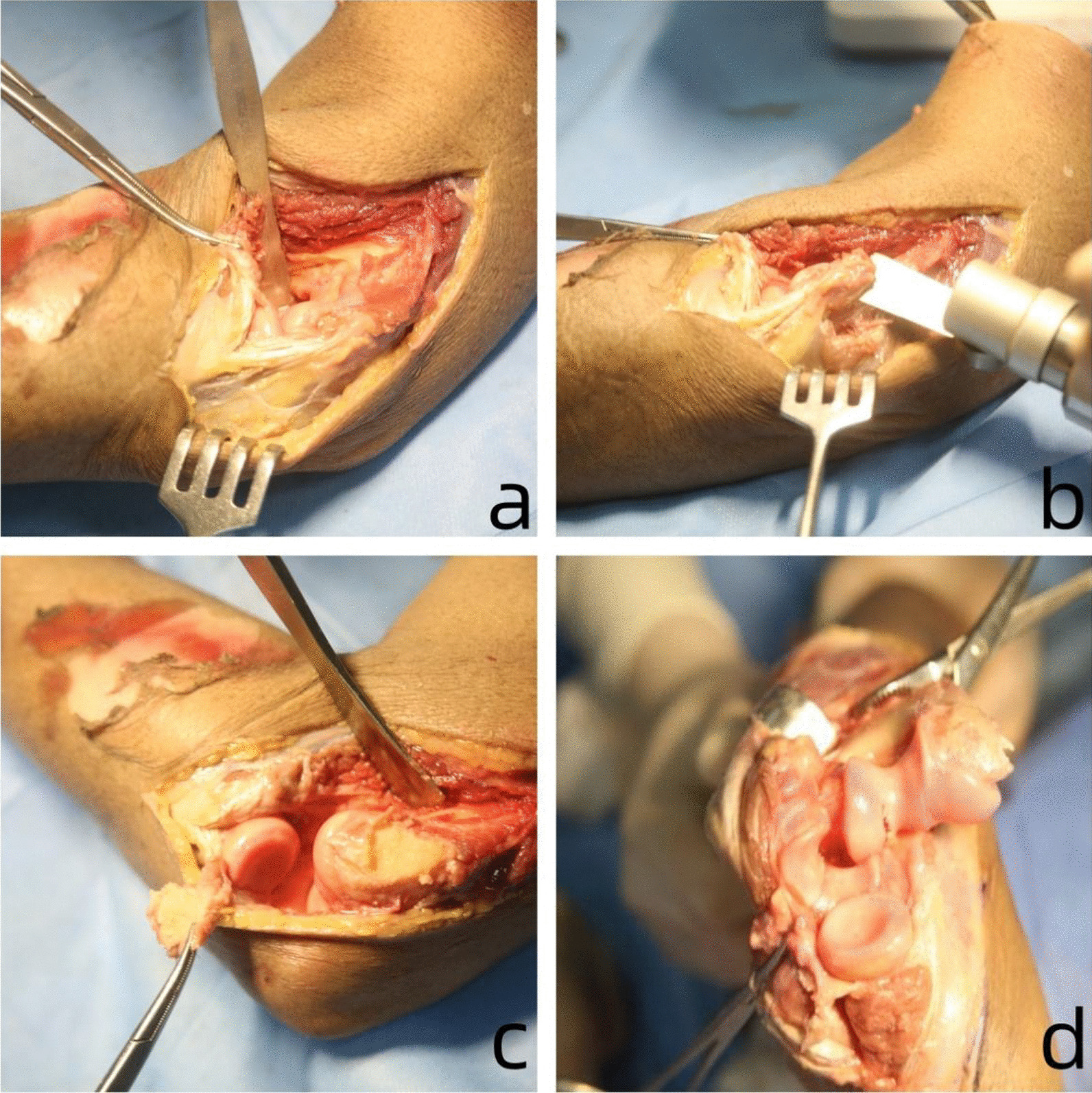
**a** The lateral collateral ligament and extensor insertion were turned distally to expose the distal articular surface of the humerus. **b** After identifying the lateral collateral ligament complex of the elbow joint, a pendulum saw was utilized to perform an osteotomy on the lateral epicondyle of the humerus. **c** The distal humeral surface was exposed after the release of the lateral collateral ligament complex. **d** The elbow joint demonstrates partial flexion and inversion, with the medial axis resembling a rotational hinge axis. The anterior and lateral dislocation of the distal humerus without tension resulted in complete exposure of the distal articular surface

**Fig. 2:**
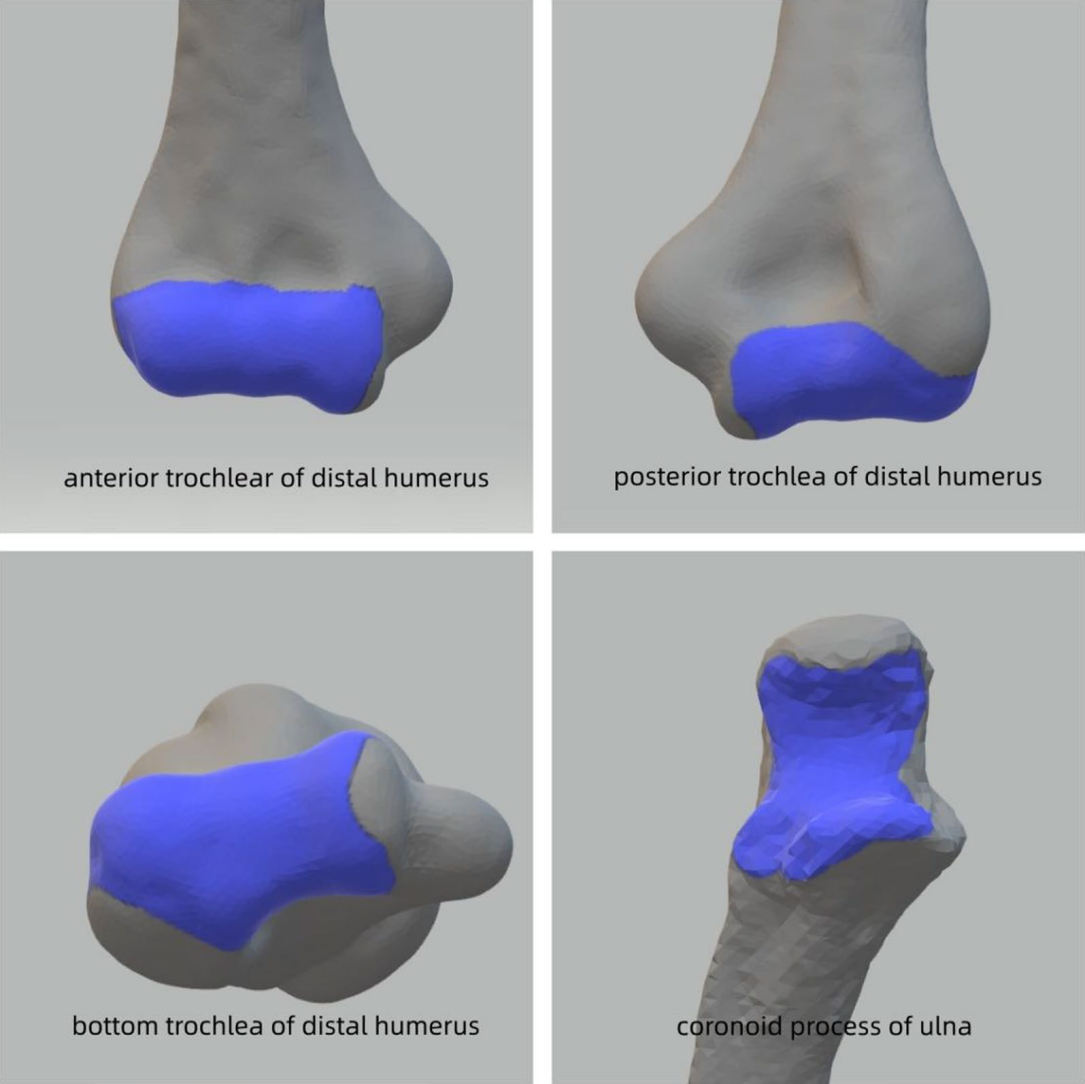
The exposed area of the elbow dislocation approach

After identifying the lateral collateral ligament complex of the elbow joint, a pendulum saw was utilized to perform an osteotomy on the lateral epicondyle of the humerus, maintaining a parallel angle to its original position and keeping approximately 4 cm away from the elbow joint space. Utmost caution should be exercised to prevent any potential harm to both the lateral collateral ligament complex and radial head during this surgical procedure. The lateral collateral ligament and extensor insertion were turned distally to expose the distal articular surface of the humerus. The elbow joint demonstrates partial flexion and inversion, with the medial axis resembling a rotational hinge axis. Furthermore, there is an anterior and lateral dislocation of the distal humerus without tension, resulting in complete exposure of the distal articular surface (Figs. 1 and 2).

### Olecranon osteotomy surgical technique

**Fig. 3:**
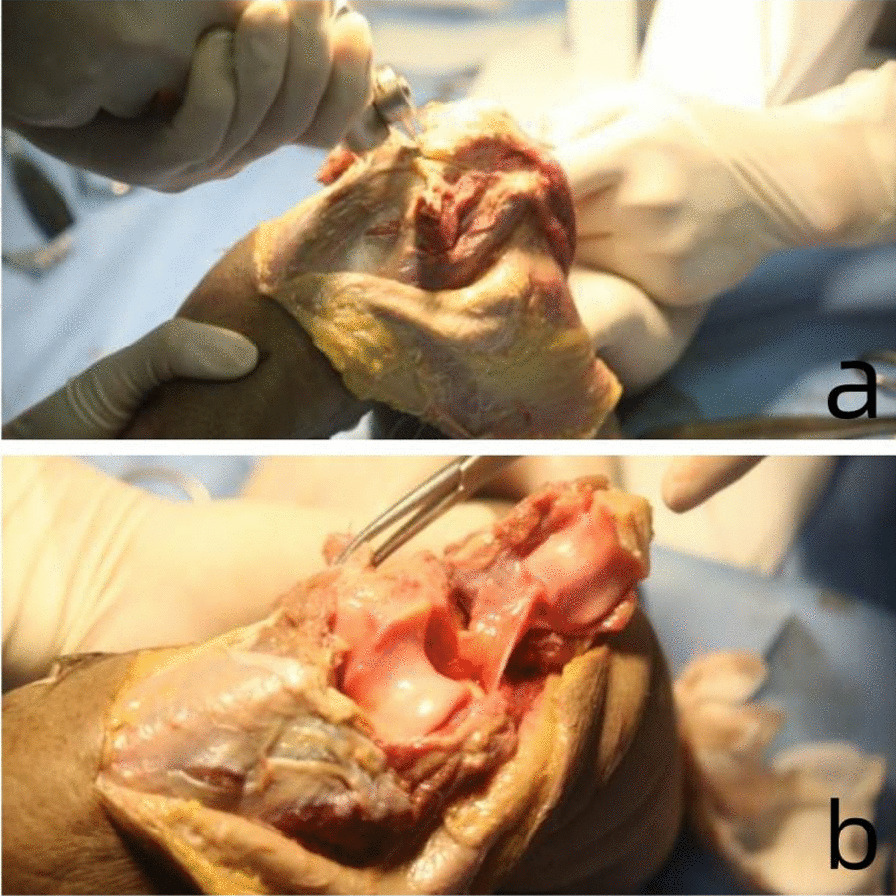
**a** Lift the muscle flap from the bilateral space of the triceps and performed a “V”-shaped osteotomy at a distance of 2 cm from the tip of the olecranon of the ulna. **b** After the osteotomy, the distal humeral surface was exposed

**Fig. 4:**
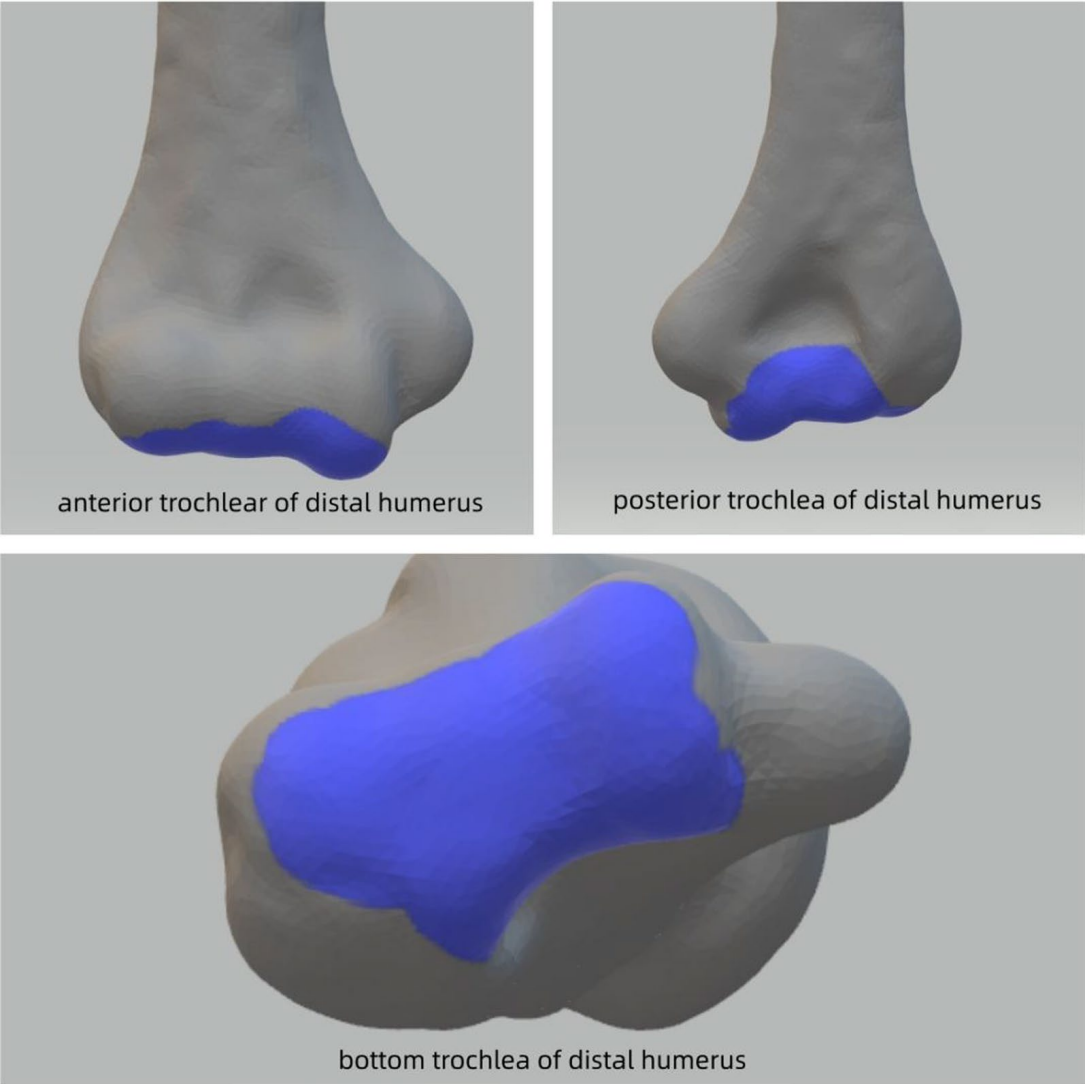
The exposed area of the olecranon osteotomy approach

After the cadaver specimen was fully thawed, the elbow was bent 90° and placed on the side edge of the autopsy table. The skin incision mark line was drawn with an oil-based marker. The skin incision was centered on the tip of the olecranon of the ulna, and a posterior midline skin incision was made, extending 5 cm at both ends, resulting in a surgical incision length of 10 cm. The skin and subcutaneous fascia should be incised, with careful dissection both internally and externally to ensure adequate exposure while protecting the ulnar nerve. The purpose of exposing the ulnar joint and distal humerus is to lift the muscle flap from the bilateral space of the triceps. Then, a “V”-shaped osteotomy was performed at a distance of 2 cm from the tip of the olecranon of the ulna. The osteotomy process was careful to avoid damage to the articular cartilage (Figs. 3 and 4).

### Measurement index

After the completion of exposure, high-resolution photographs of the humerus’ anterior and posterior articular surfaces were captured using a SONY a5100 camera. Following the completion of the joint surface exposure procedure at each approach, methylene blue stain was applied to demarcate the maintained exposure area. A 100 mm length graduated scale was positioned adjacent to the subject and captured by a camera. The ImageJ software (version 1.52a, National Institutes of Health) automatically recognized and calibrated the scale, enabling measurement of the area and proportions of all marked articular surfaces in the elbow joint. To enhance the visibility of the distinct articular surfaces, the images were processed using Photoshop (version 2022, Adobe Systems) and are presented in Figs. 2 and 4.

The proportional area method was utilized to measure the anterior distal humeral surface, the posterior distal humeral surface, the total distal humeral surface, the capitulum surface and the coronoid process facet.

### Statistical methods

The measured data were processed using SPSS 19.0 statistical software. The data in each group presented non-normal distribution with unequal variances were expressed as a median (min, max). The two-sample K-S test was used to compare between groups, with a significance level (α) set at 0.05. Statistical significance was considered when *P* < 0.05.

## Results

The median area of the distal articular surface of the humerus in the elbow dislocation group was 18.2 cm^2^, accounting for 98.2% of the total articular surface. In contrast, the olecranon osteotomy group had a median distal surface area of 11.3 cm^2^, representing 62.0% of the total articular surface area (*P* < 0.001). In the dislocation group, the median articular surface area of the humeral capitulum was 8.1 cm^2^, accounting for 100% of total humeral capitulum area, while in the olecranon osteotomy group, it was 2.9 cm^2^ representing 32.4% (*P* < 0.001). The median surface area of the humeral trochlear articular surface exposed in the dislocation group was 12.1 ± 0.8 cm^2^, accounting for 93.2% of the total humeral trochlear articular surface area, whereas in the olecranon osteotomy group it was measured 7.4 cm^2^, representing 72.5% of the humeral trochlear articular surface (*P* < 0.001). In the dislocation group, the median area exposed to the anterior trochlear articular surface of the humerus was 5.4 cm^2^, accounting for 96.0% of its total surface, whereas in the olecranon osteotomy group, it was 2.4 cm^2^, accounting for 50.3%, respectively (*P* < 0.001). The median area of the posterior trochlear articular surface exposed in the dislocation group was 6.1 cm^2^, accounting for 95.4% of the humeral posterior trochlear articular surface. In the olecranon osteotomy group, the median exposed area was 4.2 cm^2^, accounting for 100% of the humeral posterior trochlear articular surface (*P* = 0.76). The dislocation group exposed 2.6 cm^2^ for 71.3% of the ulnar coronoid process, while the ulnar coronoid process surface were not exposed at the olecranon osteotomy group (*P* < 0.001) (Table 1).

**Table 1:** Comparison of the exposed articular surface area between the elbow dislocation approach and the olecranon osteotomy approach

	Elbow dislocation group	Olecranon osteotomy group	*p*
Exposed area of distal humerus surface (%)	98.2 (97.6, 99.6)	62.0 (58.3, 64.5)	< 0.001
Exposed area of capitulum of distal humerus surface (%)	100	32.4 (28.0, 39.2)	< 0.001
Exposed area of trochlea of distal humerus surface (%)	93.2 (90.1, 96.9)	72.5 (65.2, 78.8)	< 0.001
Exposed area of anterior trochlear of distal humerus (%)	96.0 (93.0, 97.4)	50.3 (43.6, 59.1)	< 0.001
Exposed area of posterior trochlea of distal humerus (%)	95.4 (93, 100)	100	0.76
Exposed area of the articular surface of the coronoid process of ulna (%)	71.3 (66.0, 74.2)	0	< 0.001

## Discussion

In this cadaveric study, we improved the elbow dislocation technique compared with the Hoyt’s method. The approach we depicted not only provided greater exposure to the distal humeral articular surface but also a good view of the ulnar coronoid process. In addition to managing the coronal shear fractures of the distal humerus, the elbow dislocation approach can also be used on the exposure of the ulnar coronoid process fracture in the terrible triad of the elbow injury.

The typical mechanism of injury for a distal humerus shear fracture is a fall in which the elbow is extended or semi-flexed with the arm extended. The shear force displaces the capitulum forward and proximally, while the proximal end of the radius exerts shear forces on the capitulum, lateral ridge, and trochlea. When the elbow is bent from 0° to 30°, the maximum force is transmitted through the radial head [7]. The most widely used classification is the Dubberley classification. This classification classifies fractures into three types according to the degree of articular cartilage involvement. Each type is subdivided into type A or type B based on the presence or absence of posterior condylar comminution fracture [8].

The extended lateral approach is the preferred surgical approach for the treatment of distal humeral coronal fractures with open reduction and internal fixation [9]. This surgical approach provides sufficient visualization of the distal humerus articular surface and allows simultaneous management of the accompanying radial head fracture [10]. However, it is difficult to expose the olecranon wrap, especially the posteromedial fracture. For Dubberley type 2A, 3A fracture lines that extend behind the coronoid process, as well as Dubberley type B fractures characterized by partial olecranon encircle fractures, complete exposure is required to visualize the distal medial or posterior articular surface of the humerus for anatomical reduction and internal fixation under direct visualization. The ulna olecranon osteotomy approach can expose the distal humerus to the greatest extent. Among the numerous current elbow joint approaches, the ulna olecranon osteotomy approach has the largest exposure range to the distal articular surface [11, 12]. But the posterior olecranon osteotomy approach is traumatic and may lead to complications such as olecranon pain, olecranon osteotomy non-union and ulnar nerve injury. Ring et al. [5] discussed that releasing of the lateral ligamentous structures or performing osteotomy of the lateral epicondyle may enhance exposure of the working area by allowing distraction of the radial head from the capitulum. Hoyt et al. [3] dislocated the distal humerus, revealing 95.9% of the anterior surface of the distal humerus and 100% of the humeral capitulum. In our method, by utilizing flexion and inversion of the elbow, specifically through medial hinge rotation axis, the distal humerus can be rotated forward and laterally without tension. This technique allows for complete exposure of the distal articular surface of the fracture, enabling precise reduction and internal fixation under direct visualization. In Li’s study [13], a single lateral epicondyle osteotomy approach exposed 80% of the distal articular surface of the humerus, 100% of the capitulum of the humerus, 60% of the trochlea, 70% of the anterior trochlea, and 50% of the posterior trochlea. After improving dislocation in our study, the exposure of each part of the distal humerus could be achieved for over 90%.

Coronoid process fracture is caused by elbow joint dislocation or posterior lateral rotational force, while anterior medial articular surface fracture is due to excessive inward rotational force [14]. An isolated coronoid tip fracture is relative rare and indicate a symptom of dislocation or subluxation injury that spontaneously reduced [15]. For many occasions, coronoid fracture are accompanied with radial head fracture, and posterior elbow dislocation, termed as the “terrible triad of the elbow” [16]. After conducting in-depth research, O’Driscoll et al. [17] proposed a new classification system and provided treatment recommendations based on a deeper understanding of the mechanisms of injury, as well as considering the direction and morphology of fracture fragments. The anatomic classification system pertains to three primary segments of the coronoid process, namely the tip, the anteromedial facet, and the base. Adams described coronoid fractures into five common patterns tip-type fracture, mid-transverse type fracture, basal type fracture and oblique type fracture including an anteromedial type fracture and an anterolateral type fracture [18]. The method described in this study can expose approximately 70% of the surface and reveal the distribution of main fracture lines. However, the anteromedial facet of the coronoid process is not exposed as shown in Fig. 2. Therefore, a medial approach is still required for tip-type isolated coronoid fractures, which account for 29% of all coronoid fractures.

The elbow dislocation method has several important considerations. Firstly, The stability of the elbow joint needs to be reconstructed after the operation due to the osteotomy approach involving elbow dislocation, which results in varying degrees of damage to its stability, making it the highest degree of instability. Secondly, the blood supply of the lateral column of the distal humerus is mainly supplied by the posterior segmental vessels, while the blood supply of the medial column is provided by both anterior and posterior segmental vessels [19]. Therefore the posterior attached tissue should be preserved as much as possible to protect the blood supply during the operation. In the study of Mighell et al. [4], about 17% of postoperative patients had avascular necrosis of bone. But even if avascular necrosis of bone occurred, the clinical outcome including motion, strength, stability and pain remained improved. In addition, it is necessary to maintain pronation of the patient’s forearm during surgery to protect the posterior interosseous nerve.

The main limitation of this study is that the cadaveric model cannot completely represent clinical value for reasons like the gap between cadaver specimens and the articular surface in clinical practice or the statistics error in visualizing the articular surface. The mobile fragments in live surgery would complicate the exposure and largely reduce the actual technique effect. The outcomes and complications of the surgical technique need to be assessed in the clinical operation. Another limitation is the limited number of cadaver specimens available for statistical analysis of non-normal distribution, which could affect the accuracy of measurement.

## Conclusion

For complex elbow fractures, the technique of elbow dislocation provides complete exposure of the distal humerus surface and a significant portion of the coronoid process surface, facilitating direct visualization for reduction and fixation.

## Data Availability

Data are available on a supplementary file, and also, datasets used and analyzed during the current study are available from the corresponding author upon reasonable request.
